# Complexity of PEComas: Diagnostic approach, molecular background, clinical management

**DOI:** 10.1007/s00292-019-0612-5

**Published:** 2019-07-15

**Authors:** K. Utpatel, D. F. Calvisi, G. Köhler, T. Kühnel, A. Niesel, N. Verloh, M. Vogelhuber, R. Neu, N. Hosten, H.-U. Schildhaus, W. Dietmaier, M. Evert

**Affiliations:** 1 Institute of Pathology, University of Regensburg, Franz-Josef-Strauß-Allee 11, 93053 Regensburg, Germany; 2 Department of Gynecology, University Medical Center Greifswald, Greifswald, Germany; 3 Department of Otorhinolaryngology, University Hospital Regensburg, Regensburg, Germany; 4 Department of Gynecology, Preetz Hospital, Preetz, Germany; 5 Department of Radiology, University Hospital Regensburg, Regensburg, Germany; 6 Department of Internal Medicine III, Hematology and Oncology, University Hospital Regensburg, Regensburg, Germany; 7 Department of Thoracic Surgery, University Hospital Regensburg, Regensburg, Germany; 8 Department of Radiology, University Medical Center Greifswald, Greifswald, Germany; 9 Institute of Pathology, University Medical Center Essen, Essen, Germany

**Keywords:** Genetic translocation, Immunohistochemistry, Lymphangioleiomyomatosis, Perivascular epithelioid cell neoplasms, TOR serine-threonine kinases, Genetische Translokation, Immunhistochemie, Lymphangioleiomyomatose, Perivaskuläre epitheloidzellige Tumoren, TOR-Serin-Threonin-Kinasen

## Abstract

Perivascular epithelioid cell neoplasms (PEComas) are a family of mesenchymal neoplasms with features of both melanotic and smooth muscle differentiation. PEComa morphology is highly variable and encompasses epithelioid to spindle cells often with clear cytoplasm and prominent nucleoli. Molecularly, most PEComas are defined by a loss of function of the TSC1/TSC2 complex. Additionally, a distinct small subset of PEComas harboring rearrangements of the *TFE3* (Xp11) gene locus has been identified. By presenting a series of three case reports with distinct features, we demonstrate diagnostic pitfalls as well as the importance of molecular work-up of PEComas because of important therapeutic consequences.

## Abstract (de)

Perivaskuläre epitheloidzellige Tumoren (PECome) gehören zu einer Familie von mesenchymalen Neoplasien mit Merkmalen der melanotischen und glattmuskulären Differenzierung. Die Morphologie der PECome ist sehr variabel und umfasst epitheloide und spindelige Zellen, oft mit klarem Zytoplasma und prominenten Nukleoli. Molekular sind die meisten PECome durch einen Funktionsverlust des TSC1-TSC2-Komplexes definiert. Zusätzlich wurde eine kleine Untergruppe von PEComen identifiziert, die Rearrangements des *TFE3*(Xp11)-Genlocus aufzeigen. Anhand von 3 Fallberichten sollen die diagnostischen Fallstricke und die Bedeutung der molekularen Charakterisierung von PEComen auch wegen der therapeutischen Konsequenzen näher dargestellt werden.

Perivascular epithelioid cell neoplasms (PEComas) are a family of mesenchymal neoplasms with features of both melanotic and smooth muscle differentiation that are thought to derive from distinctive perivascular epithelioid cells. Angiomyolipoma of the kidney, lymphangiomyomatosis, and clear cell “sugar” tumor of the lung are prototype members of this tumor family.

Other PEComas occur in many anatomic sites such as the uterus, liver, and pancreas [11, 17, 29]. Angiomyolipoma and lymphangiomyomatosis are far more common in women and known to be associated with tuberous sclerosis. Most other PEComas develop spontaneously [10, 12]. The morphology of PEComas is highly variable and encompasses epithelioid to spindle cells often with clear cytoplasm and prominent nucleoli. Genuine melanin pigment can also be detected in a subgroup of cases [22]. Owing to the morphological diversity and depending on the tumor location, many differential diagnoses have to be considered including malignant melanoma, carcinomas, sarcomas, and smooth muscle tumors (Table 1).

**Table 1:** Immunohistochemical profile of PEComa and important differential diagnosis

	TFE3	S100	Melan A	HMB-45	MITF	Sm-Actin	Caldesmon	Desmin	Myogenin	MyoD1	Myoglobin	Pan-CK	INI1	CD10	ER	PR
*PEComa*	**−**	**−/+**	**+**	**+**	**+**	**+**	**+/−**	**+/−**	**−**	**−**	**−**	**−**	**+**	**−**	**−/+**	**−/+**
*TFE3-PEComa*	**+**	**−/+**	**+**	**+**	**+**	**−/+**	**−/+**	**−/+**	**−**	**−**	**−**	**−**	**+**	**+/−**	**−/+**	**−/+**
Melanoma	−	+	+	+	+	−	−	−	−	−	−	−	+	+/−	−	−
ASPS	+	+/−	−	−	−	−/+	−	+/−	−	−	−	−	+	−/+	−	−
Pleomorphic RMS	−	−	−	−	−	−	−	+	+/−	+/−	+	−	+	−/+	−	−
TFE3-RCC	+	−	−/+	−/+	−	−	−	−	−	−	−	−/+	+	+/−	−	−
Low-grade ESS	−	−	−	−/+	−	+/−	−	−/+	−	−	−	−/+	+	+	+	+
High-grade ESS	−	−	−	−/+	−	−	−	−	−	−	−	−	+	−	−	−
Uterine LMS	−	−	−	−/+	−	+	+	+	−	−	−	−/+	−	+/−	−/+	−/+
Epithelioid sarcoma	−	−	−	−	−	−	−	−	−	−	−	+	−	−	−	−

*PEComa *perivascular epithelioid cell neoplasm*, ASPS* alveolar soft part sarcoma, *RMS* rhabdomyosarcoma, *RCC* renal cell carcinoma, *ESS* endometrial stromal sarcoma, *LMS* leiomyosarcoma, *Sm* smooth muscle, *CK* cytokeratin

+ typically positive, +/− variable, often positive, −/+ variable, mostly negative, − typically negative

Molecularly, most PEComas, including sporadic ones, are defined by a loss of function of the TSC1/TSC2 complex, in the majority of the cases the result of a loss of heterozygosity (LOH) in the *TSC2* gene, leading to increased mTORC1 activation and deregulated cell growth signaling [5, 8, 14]. Additionally, a distinct small subset of PEComas harboring rearrangements of the *TFE3*(Xp11) gene locus have been identified. The PEComas of this group exhibit distinctive morphological features known from other TFE3 rearranged tumors such as Xp11-translocation renal cell cancer and alveolar soft part sarcoma, including an alveolar growth pattern and an epithelioid cytomorphology [2].

Here, by presenting a series of three case reports with distinct features, we demonstrate the diagnostic pitfalls that the pathologist encounters with this disease. In addition, we underline the importance of a detailed molecular analysis of PEComas to design and develop appropriate therapeutic strategies against this disease.

## Case 1

**Fig. 1:**
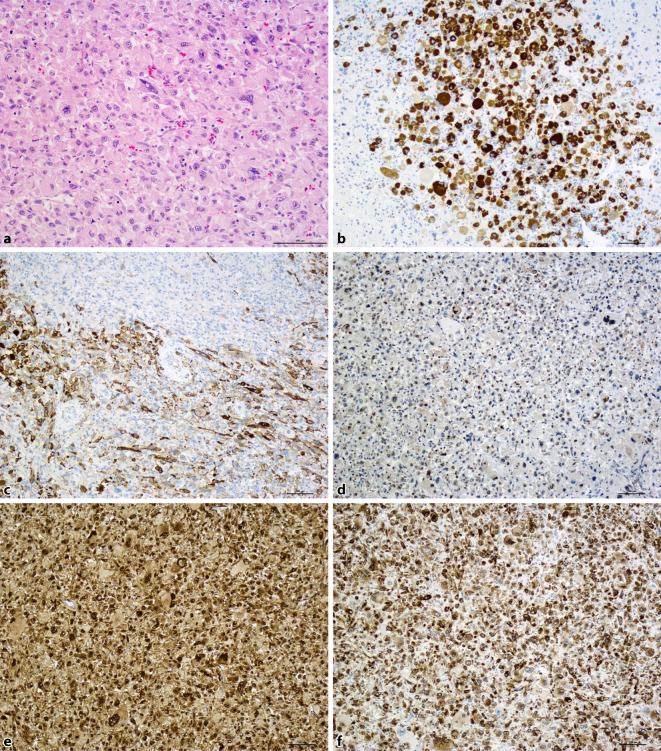
Case 1: Histological and immunohistochemical aspects. **a** H&E, ×200; **b** HMB-45, ×100; **c** caldesmon, ×100; **d** p-mTOR, ×100; **e** p-4EBP1, ×100; **f** p-RPS6, ×100

In March 2017, a 12.5-cm large lobulated tumor without necrosis was resected from the left soleus muscle of a 58-year-old patient. Histologically, the tumor revealed a solid growth pattern of pleomorphic cells with abundant granular eosinophilic cytoplasm (Fig. 1a). The nuclei showed irregular contours and prominent nucleoli. Occasionally, nuclear inclusions could be seen and on average 1–2 mitoses/HPF were found. Immunohistochemically, the tumor showed a patchy positivity for HMB-45 and caldesmon (Fig. 1b, c) as well as smooth muscle actin. Other melanocytic markers, such as Sox-10, Melan A, MITF, and S‑100 and myogenic markers, such as desmin, MyoD1, and myogenin, were both negative. Furthermore, the tumor cells were negative for cytokeratin (AE1/AE3) and CD68 (PGM-1). TFE-3 immunohistochemistry (clone MRQ-37, Cell Marque, Rocklin, CA, USA) revealed only a weak-to-moderate nuclear staining, which is not characteristic of a TFE-3 translocated tumor. In addition, a TFE-3 translocation was absent on fluorescence in situ hybridization (FISH) analysis (Zyto*Light *® SPEC TFE3 Dual Color Break Apart Probe, ZytoVision GmbH, Bremerhaven, Germany). The diagnosis of a malignant PEComa was made. Because of the narrow resection margin (0.1 cm), local radiation was performed.

**Fig. 2:**
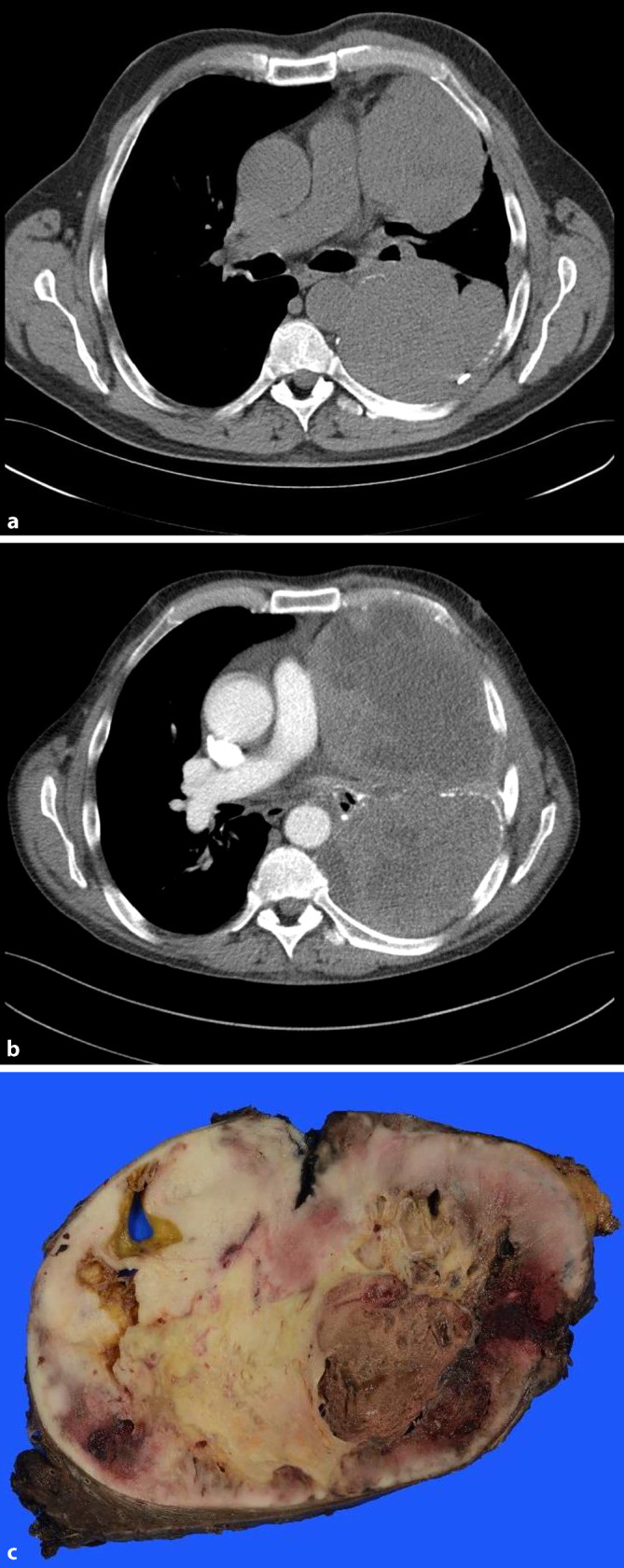
Case 1: Computed tomography scan of the thoracic relapsed tumor and macroscopy of the resected tumor. **a** Two new tumor nodules in the left thorax in July 2018. **b** Tumor progression in November 2018. **c** Resected tumor after 5‑month mTOR inhibitor treatment

In December 2017, the patient developed a left-sided pleural dissemination with tumor nodules of up to 15 cm in size and partial infiltration of the left lower lobe of the lung; the lesions were completely resected via partial pneumo- and pleurectomy. Necrosis was again not recognizable in the tumor. The patient refused the recommended postprocedural radiation. In June 2018, two new metastases (8.9 cm and 7.1 cm in diameter) in the left mediastinum were discovered on computed tomography (CT) scans and were histologically verified by biopsy (Fig. 2a). At that time, the tumor was examined immunohistochemically and molecularly in order to find possible drug targets. In a multigene panel analysis (Human Actionable Solid Tumor Panel kit, Qiagen, Venlo, The Netherlands) only a pathogenic *TP53* mutation in exon 5, but no other mutations especially no melanoma-associated mutations, could be detected. Immunohistochemically, the tumor revealed a weak-to-moderate expression of phosphorylated mTOR (protein phosphorylation site SER2884, Cell Signaling Technology, Danvers, MA, USA) and a strong expression of the two main target proteins of the mTOR complex 1, namely, phosphorylated eukaryotic translation initiation factor 4E binding protein 1 (p-4EBP1; Clone 53H11, Cell Signaling Technology) and phosphorylated ribosomal p‑RPS6 (protein phosphorylation site SER235/236, Cell Signaling Technology; Fig. 1d–f). On the basis of the immunohistochemical results, treatment with everolimus (10 mg daily), an mTOR inhibitor, was started in July 2018. On a restaging CT scan in September 2018, a mixed response was detected, which was judged as stable disease. Because of iron-deficiency anemia, fatigue, and mucositis, the dosage of the mTOR inhibitor had to be reduced to 5 mg. In November 2018, tumor progression was visible on CT scans. The metastasis in the left mediastinum measured 13.9 and 10 cm (Fig. 2b). In December 2018, a re-thoracotomy with left-sided pneumectomy, pericardial sac resection, and tumor resection was performed. The resected tumor showed large necrotic areas, accounting for about 50% (Fig. 2c), which were evaluated as effects of mTOR inhibitor therapy.

## Case 2

In January of 2016, a 39-year-old male patient presented as an emergency case because of severe epistaxis. His medical record showed an embryonal carcinoma of the testis in 2006. Clinical examination revealed a bleeding fleshy polypoid mass located at the left middle nasal concha. Computed tomography scans showed pansinusitis without a clear-cut tumor formation. No distant metastases were found on positron emission tomography (PET) scans. The polypoid tumor was subsequently resected.

**Fig. 3:**
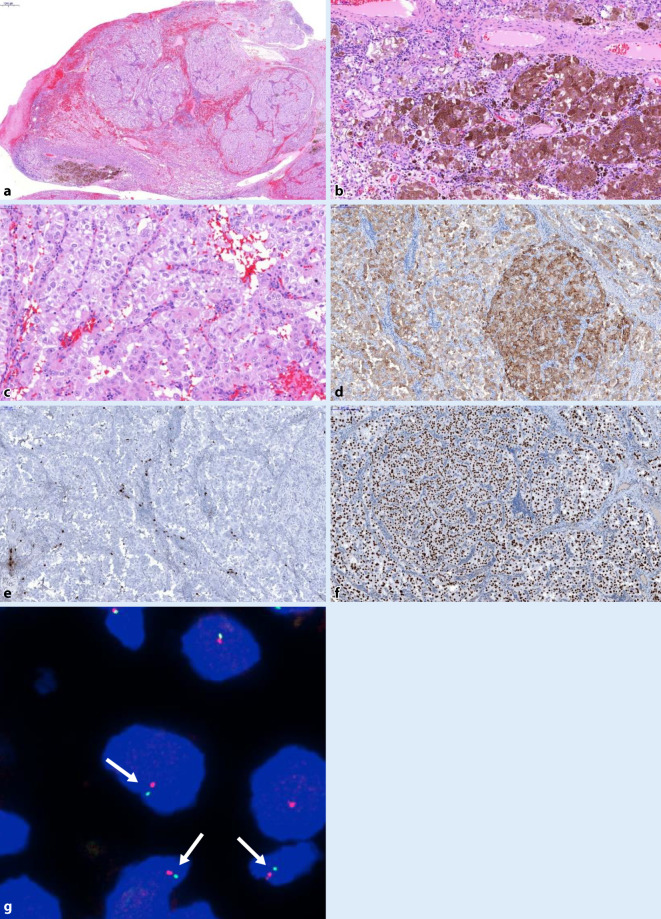
Case 2: Melanotic perivascular epithelioid cell neoplasm of the nasal cavity. **a** Overview, H&E, ×16; **b** detection of melanin, ×200; **c** Atypical tumor cells, ×100; **d** HMB-45, ×100×; **e** S-100, ×100; **f** TFE3, ×100; **g** fluorescence in situ hybridization: *TFE3* break-apart probe reveals small gaps between 3′*TFE3* and 5′ *TFE3* in 3 interphase cells (*arrows*) in a male patient, indicating the translocation of the only X chromosome

Macroscopically, we saw a soft brown–red polypoid tumor measuring up to 2.5 cm. Histological examination revealed a neoplasm composed of epithelioid cells in a prominent alveolar to nested architecture (Fig. 3a, c). The cytoplasm was clear to finely granular, the vesicular nuclei were atypical and showed prominent nucleoli. Of note, areas of the tumor cells were heavily pigmented with melanin (Fig. 3a, b). No suspicious PAS-positive inclusions were present, and mitoses, necrosis, or angioinvasion were likewise not detected. Immunohistochemically, the tumor cells were diffusely HMB-45 positive and showed a strong nuclear expression of TFE3 (Fig. 3d, f). The tumor cells were negative for any other melanocytic markers such as S100 (Fig. 3e), Melan A, MITF, SOX10, all myogenic markers (SMA, MSA, desmin, caldesmon) and cytokeratins. The Ki67-index was 5%. In line with the other results, Melanoma-associated mutations (*BRAF, NRAS, c‑Kit*) were absent and malignant melanoma was ruled out. Pursuing the nuclear TFE3 expression, FISH analysis with a *TFE3* gene break-apart probe was performed, which was initially interpreted as being negative. Only a small subset of tumor cells with minimal separation of the 5′*TFE3* and 3′*TFE3* probe signals were detected, not meeting established cut-off values. In order to verify the immunohistochemical results and to possibly unveil the binding partner, we performed RNA-sequencing (TrueSight RNA Fusion Panel, Illumina, San Diego, CA, USA), which disclosed an extremely rare *NONO–TFE3* fusion. *NONO* and *TFE3* genes are both located on the X chromosome leading to an inverse translocation. The proximity of the two genes is the reason for the only minimal signal separation and initial misinterpretation of the *TFE3* FISH analysis. In light of this specific constellation, a second FISH analysis, in which small gaps of one to two signal diameters were now correctly interpreted, confirmed the *TFE3* gene disruption (Fig. 3g). Finally, the diagnosis of a melanotic *TFE3*-(Xp.11)-rearranged PEComa was made. The follow-up of the patient remained unremarkable 2 years after resection.

## Case 3

**Fig. 4:**
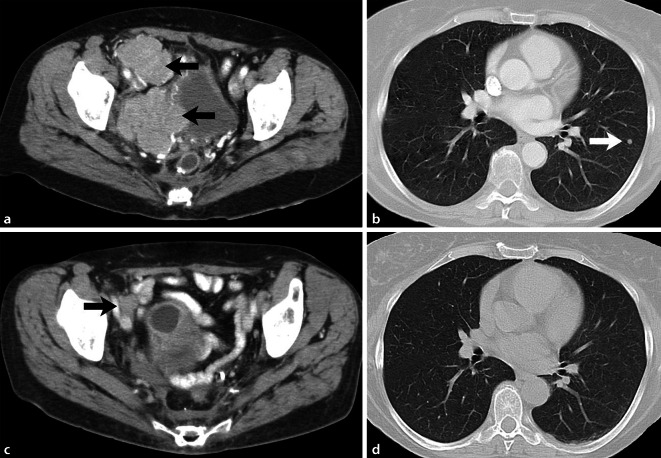
Case 3: Contrast-enhanced computer tomography before everolimus treatment and a follow-up scan 4 months after initial treatment. **a** Large tumor detected on the right side in the pelvis (*black arrows*); **b** lung metastasis (*white arrow*); **c** detection of a small scarred residual tumor 4 months after treatment (*black arrow*); **d** no lung metastasis can be observed

**Fig. 5:**
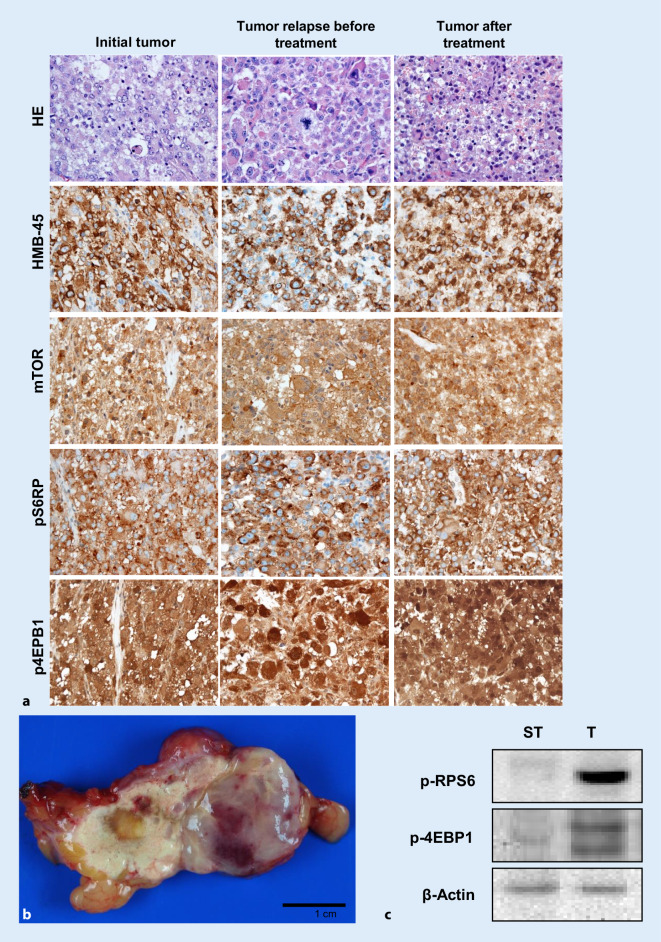
Case 3: Malignant perivascular epithelioid cell neoplasm of the uterus.** a** H&E, HMB-45, p‑mTOR, p‑4EBP1, and p‑RPS6, ×400. **b** Macroscopy of the residual pelvic tumor after 4 months of mTOR inhibitor treatment. **c** Western blot analysis of mTOR downstream effectors p‑RPS6 and p‑4EBP1 in the tumor (*T*) and non-tumorous surrounding tissue (*ST*). β‑Actin was used as a loading control. Strong induction of the two mTOR effector proteins in the tumor part

A 58-year-old woman underwent a hysterectomy in December 2010 in an external hospital under the suspicion of having uterine leiomyomas. Histologic analysis of the morcellated uterus, however, led to the diagnosis of an epithelioid, possibly malignant, PEComa of the uterus. In the follow-up examination 3 months later, the patient presented with a multinodular large recurring intraperitoneal pelvic tumor, located in the peritoneum of the cecum, sigmoid/descending colon, greater omentum, mesorectum, bladder and retroperitoneally near the right ureter. All visible tumor nodules were resected in April 2011. However, only 2 months later, a new multinodular pelvic relapse and, in addition, pulmonary metastases (Fig. 4a, b) were noted. In August 2011, the patient was transferred to the German Clinical Competence Center for Sarcomas and Mixed Tumors of the Female Genital Tract in Greifswald. Because the pelvic tumor, measuring 15 cm in diameter, was not resectable and the disease had become systemic with four pulmonary metastases in both lungs, a (palliative) chemotherapeutic approach was considered as the appropriate treatment. At that time, case reports showed that mTOR-targeted therapy could be of value because this signaling pathway is usually strongly active in this tumor type [9, 13, 25, 27]. Of note, retrospective analyses of these case reports have shown that tumors proven to be mTOR-active by immunohistochemistry are particularly prone to regression [13, 27]. Thus, a biopsy of the pelvic tumor was performed to examine the activity of mTORC1-signaling as a biomarker for subsequent therapy. Histologically, the tumor showed the typical morphology and immune profile of an epithelioid malignant PEComa, i.e., co-expression of myogenic and melanocytic markers, such as desmin, Melan-A, and HMB45. Of note, the tumor revealed a strong immunohistochemical expression of not only mTOR but also its two main target proteins of the mTOR complex 1 by use of phosphorylation-specific antibodies against the active forms of the proteins, i.e., p‑4EBP1 and p‑RPS6. We also examined the primary tumor regarding mTOR activity retrospectively and found the same pattern of alterations (Fig. 5a). These tissue biomarkers provided the scientific basis in this individual case to start everolimus therapy, an mTOR inhibitor. The patient received everolimus in a daily dose of 10 mg for four consecutive months until December 2011. At the end of therapy, the patient complained mainly of severe fatigue, a known side effect of the therapy.

Everolimus therapy was extremely successful. Imaging studies showed a complete regression of the pulmonary metastases and an astonishing shrinkage of the pelvic tumor bulk from 15 cm to 3 cm in diameter (Fig. 4c, d). As this success proved to be much better than expected, the initial approach from a palliative systemic therapy was switched to a neoadjuvant approach that was followed by the resection of the residual tumor nodule in January 2012. The 3‑cm-large tumor was resected in total and consisted of 50% vital tumor tissue (Fig. 5b) with identical histology and immune phenotype as in the prior manifestations (Fig. 5a). In necrotic areas, a strong resorptive inflammatory reaction by foamy macrophages and histiocytic giant cells was noted, intermingling with single or small clusters of damaged but vital tumor cells. Of note, the vital tumor tissue still showed strong activation of mTORC1 signaling on immunohistochemistry and also by immunoblotting of frozen tumor tissue, which has never been investigated before (Fig. 5c). As the patient was now completely free of disease, and since no data about a beneficial continuous pharmacological therapy were available, the local tumor board decided to discontinue mTOR inhibitor therapy. However, 5 months later, the patient presented with another tumor relapse, showing a large non-resectable tumor mass in the pelvic region and the right kidney as well as new lung metastases. Since the tumor still showed high mTOR activity in the last resection, the everolimus treatment was once again started. Again, the tumor showed an impressive response to the targeted therapy, lasting for about 1 year. However, in June 2013, the pelvic recurrence and the lung metastasis showed tumor progression and everolimus treatment was discontinued and switched to conventional therapy with doxorubicin monotherapy (six cycles 60 mg/m^2^), which was of limited benefit. The patient died in 2014 of brain metastasis 3 years after the initial diagnosis. An autopsy was not performed.

## Discussion

Perivascular epithelioid cell neoplasms are rare tumors occurring in many anatomic sites and, depending on the location, leading to distinct differential diagnosis. Thus, awareness of this entity is mandatory in order to arrive at the correct diagnosis, and the investigation of the molecular background may help to predict the response to targeted therapies.

### Diagnostic approach

Perivascular epithelioid cell neoplasms are a rare group of tumors with putative origin in perivascular epithelioid cells and usually a combined myogenic and melanocytic immunophenotype. Although most PEComas—particularly the ones that have been known for a long time under different names in specific organs, such as angiomyolipomas of the kidney or pulmonary lymphangioleiomyomatosis—behave mostly in a benign manner, particularly epithelioid PEComas in other organs can also behave unpredictably in a malignant way [11]. High-risk features such as a size of ≥5 cm, infiltrative growth pattern, high nuclear grade and cellularity, mitotic rate of ≥1/HPF, as well as necrosis and vascular invasion were proposed by Folpe and Mentzel [11]. Bleeker has proposed an update for a malignant classification demonstrating that only a size of ≥5 cm and a mitotic rate of ≥1/HPF are significantly associated with potential malignant behavior und recurrence [7]. Consistent with this classification, all three cases presented here could be correctly labeled as benign or malignant. In addition, as in our first case, the molecular detection of a *TP53* mutation could also be used to predict malignant behavior [6].

The differential diagnostic spectrum varies particularly because of the wide range of tumor locations. Concerning our first case (lower leg tumor), differential diagnoses such as pleomorphic rhabdomyosarcoma or leiomyosarcoma, epithelioid sarcoma, malignant granular cell tumor (incorrect external primary diagnosis) and malignant melanoma were considered. Pleomorphic rhabdomyosarcoma shows at least focally an expression of skeletal muscle-specific markers, i.e., myoglobin, MyoD1, or myogenin, and lacks positivity for melanocytic markers. Leiomyosarcoma exhibits positivity for smooth muscle markers but lacks positivity for melanocytic markers. Epithelioid sarcoma typically displays a lack of INI1 expression, and malignant granular cell tumor expresses S100. All differential diagnoses could be ruled out by demonstrating a co-expression of myogenic and melanotic markers.

Regarding our second case (nasal cavity), malignant melanoma was our first approach. Malignant melanoma and PEComa of the nasal mucosa share many clinical, morphological, and immunohistochemical features, such as a bleeding polypoid mass, epithelioid morphology, and expression of melanocytic markers. The strong expression of HMB-45 in this pigmented neoplasm would lead to the diagnosis of a malignant melanoma. However, the absence of any other melanotic marker especially SOX-10 and MITF, the absence of a relevant mitotic activity, and the prominent eosinophilic to clear cytoplasm of the tumor cells are clues to look for other tumor types. Since the tumor also showed an alveolar growth pattern, alveolar soft part sarcoma with aberrant melanin pigmentation had to be considered. The strong TFE3 immunohistochemistry expression also fits this diagnosis. However, typical rod-shaped intracytoplasmic inclusions could not be demonstrated on PAS stain after diastase digestion. The diagnosis was finally excluded by showing an intact *EWSR1* gene via FISH analysis (Vysis EWSR1 Break Apart FISH Probe Kit, Abbott Laboratories, Chicago, IL, USA) as well as RNA-sequencing. After excluding all other differential diagnoses, we rendered the diagnosis of a *TFE3-*translocated pigmented PEComa. This subtype of PEComas differs from conventional PEComas to the extent that it consists of an epithelioid phenotype and attenuated or missing expression of myogenic markers [2]. Thus, PEComa should be considered as a differential diagnosis of a pigmented lesion in mucosal sites.

Regarding our third case (uterus), the differential diagnosis included leiomyosarcoma and high-grade endometrial stromal sarcoma; in cases of a more spindle-cell PEComa, low-grade endometrial stromal sarcoma can also be considered. Co-expression of smooth muscle markers and CD10 together with negative melanocytic markers favors the diagnosis of leiomyosarcoma. Although all the considered tumor types can be positive for sex hormone receptors, an intense estrogen receptor immunolabeling is more often found in low-grade endometrial stroma sarcoma. In addition, low-grade endometrial stroma sarcoma harbors specific chromosomal rearrangements involving *JAZF1* and *PHF1* [19, 20]. High-grade endometrial stroma sarcomas show a strong and diffuse cyclin D1 positivity, are usually negative for CD10 and hormone receptors as well as melanocytic markers, and harbor the *YWHAE–FAM22* genetic fusion in most cases [15, 16]. An important pitfall to be aware of is that endometrial stromal sarcoma, leiomyosarcoma, and even leiomyoma can occasionally show expression of HMB-45. Mostly, the reported expression of HMB-45 was found focally in a subset of tumor cells [1, 23]. Regarding the diagnosis of leiomyosarcoma, this immune profile can be indistinguishable from that of a PEComa. In difficult cases, immunolabeling for more than one melanocytic marker and the demonstration of *TSC2* mutations/alterations or *TFE3* translocations might be helpful to differentiate PEComa from aberrant HMB-45-positive leiomyosarcoma.

In summary, PEComas usually co-express myogenic and melanocytic markers. In terms of melanocytic differentiation, HMB-45 and Melan A are the most commonly expressed markers. S100 is rarely and, if so, then focally detectable with varying intensity. The most frequently expressed myogenic marker is smooth muscle actin. Desmin or caldesmon expression is observed in about 30% of cases, especially in sclerotic PEComas. In general, pronounced epithelioid PEComas tend to show a stronger expression of melanocytic markers than myogenic markers, and predominately spindle-cell PEComas show an opposite expression profile. There are no cut-offs for a diagnostically necessary proportion of positive cells or for the intensity of the immunohistochemical staining. With moderate or strong expression and otherwise appropriate findings, even a single melanocytic marker is sufficient for the diagnosis, and myogenic differentiation may be completely absent, especially in *TFE3*-translocated PEComas. In case of doubt, molecular genetic clarification should be sought.

### Molecular background

Molecularly, most PEComas, including sporadic ones, are defined by a loss of function of the TSC1/TSC2 complex, in the majority of the cases the result of a loss of heterozygosity (LOH) in the *TSC2* gene, leading to increased mTORC1 activation and deregulated cell growth signaling [5, 8, 14]. Additionally, a distinct small subset of PEComas harboring rearrangements of the *TFE3*(Xp11) gene locus have been identified. This subgroup also has no association with tuberous sclerosis [2]. TFE3, a transcription factor belonging to the microphthalmia (MIFT/TFE) transcription factors along with TFEB, TFEC, and MITF, triggers the Met receptor tyrosine kinase by direct transcriptional upregulation, leading to an activation of the downstream pathways, such as the PI3K/AKT/mTOR pathway [21, 26].

Argani et al. investigated almost 1500 tumors of 64 histologic tumor types from 16 sites and found TFE3 nuclear immunoreactivity to be restricted to alveolar soft part sarcoma (19/19 cases) and some distinct types of renal cell carcinoma (20/21 cases). Only in six other cases in their series was a strong or moderate expression of TFE3 observed (two adrenal cortical carcinomas, two granular cell tumors, one bile duct carcinoma, and one myxofibrosarcoma; [3]). A few years later, the authors also found that a distinctive subtype of PEComa harbors *TFE3* gene fusions [2]. This raises some interesting questions about the nature and relationship of these various tumors, particularly imposing the hypothesis of a molecularly defined but otherwise heterogenous tumor group. It also reinforces the impression that there is often a close correlation between morphology and genetics in human tumors, demonstrated here by the fact that all *TFE3*-positive tumor types share several microscopic features, such as an alveolar/nesting growth pattern of large eosinophilic cells with clear cytoplasm.

Additionally, we present here a rare TFE3-translocated PEComa with a *NONO–TFE3 *fusion. To date, this specific fusion has been described in fewer than 20 renal cell carcinomas, a Xp11 neoplasm of the prostate, one melanotic PEComa of the orbita, and the only other published melanotic PEComa of the sinonasal mucosa apart from this case [4, 18, 28, 30]. Interestingly, three of the extrarenal cases including ours are located in the sinonasal/orbital region, suggesting that this anatomical region might be typical for this molecularly distinct and rare subtype.

**Fig. 6:**
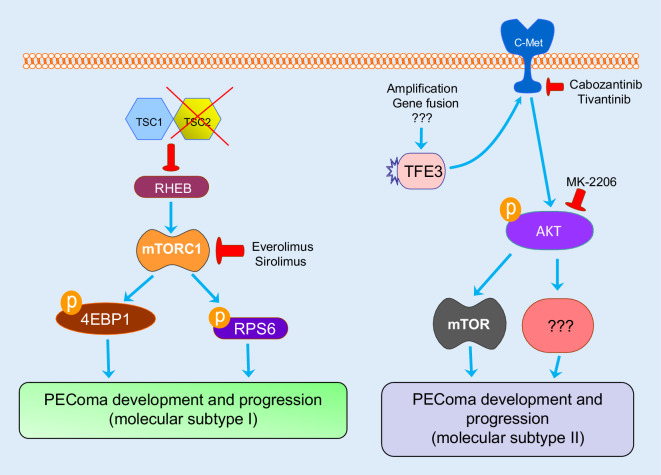
Scheme depicting the two main molecular subgroups of perivascular epithelioid cell neoplasms (PEComa) identified to date. In the first subtype, the most frequent type, loss of function of the complex consisting of TSC Complex Subunit 1 and 2 (*TSC1* and *TSC2*) proteins leads to activation of the Ras homolog, mTORC 1 binding protein (*RHEB*), with consequent induction of mTORC1. Once activated, mTORC1 triggers the activation of its downstream targets, S6 ribosomal protein (*RPS6*) as well as the inhibition of the tumor suppressor 4EBP1, thus leading to unrestrained cell growth. mTORC1-driven events are mediated by phosphorylation (*p*) mechanisms. In the second PEComa molecular subtype, various molecular mechanisms (amplifications, gene fusion, etc.) induce elevated transcriptional activity of TFE3, resulting in activation of the c‑Met proto-oncogene and induction of the downstream effector proteins, which are partly known (*AKT, mTOR*) and partly unknown (*???*), harboring important pro-oncogenic and growth properties. Of note, many of the inducers and effectors described can be specifically inhibited (as indicated by *red, blunted arrows*) by available drugs

Altogether, the available data clearly indicate the existence of at least two main molecular subtypes of PEComas, with the first subtype consisting of unrestrained activation of the mTORC1 pathway, while the second subgroup presents an elevated transcriptional activity of TFE3 and subsequent induction of pro-oncogenic pathways (c-Met, AKT, mTOR). The molecular mechanisms underlying tumor development in the two PEComa subtypes are represented in Fig. 6. Beside the usefulness of these molecular difference for tumor classification, such distinction might be highly helpful for the development of tailored therapy against this disease.

### Clinical management

The important study by Kenerson revealed that PEComas usually show increased mTOR signaling, in most cases related to impairment of TSC2 function [14]. Since mTOR signaling can be regarded as the tumor driver in these cases, its inhibition represents a promising target for pharmacologic therapy. Although some reports have shown beneficial effects of drugs directed against mTOR, others have not and some authors have doubted the general importance of this therapy [9, 24, 25, 27]. It appears that the combination of genetic findings in the TSC1/TSC2 complex and staining of the mTOR signaling pathway may predict response to mTOR inhibitors. We agree with Subbiah et al. that these rare tumors are heterogeneous and may possibly never be examined in a large clinical trial [25]. However, this should still be regarded as the best possible approach in the future, obviously including the need of many participating centers, combining their small individual number of cases in a standardized manner, especially for unravelling the mechanisms of resistance to mTOR inhibition that are still unknown.

Up to this point, in our opinion, the clinicopathological work-up of cases 1 and 3 illustrates the best therapeutic approach for patients suffering from a metastatic PEComa. In line with many other examples in which biomarkers have a predictive value for individualized therapy strategies, such as steroid hormone expression in breast cancer or mutational analysis of tyrosine kinases in numerous tumor entities, malignant PEComas, too, should be examined for biomarkers that indicate an increased mTOR activity to obtain a scientific robust rationale to start mTOR inhibition therapy. As long as no prospective study has been undertaken, the analysis of p‑4EBP1 and p‑RPS6 seem to be a suitable procedure.

## Practical conclusion

- Perivascular epithelioid cell neoplasms (PEComas) are a mesenchymal neoplasm with myogenic and melanotic differentiation.
- A size of ≥5 cm and a mitotic rate of ≥1/HPF are significantly associated with potential malignant behavior and recurrence.
- Two molecular subtypes of PEComas have been identified: The first subtype is defined by a loss of TSC1/2, while the second subgroup harbors *TFE3* rearrangements.
- The first molecular subtype shows unrestrained activation of the mTORC1 pathway as a possible therapeutic target with mTOR inhibitors.
- *TFE3*-translocated PEComas are characterized by an epithelioid phenotype and attenuated or missing expression of myogenic markers.
